# Training the trainees in robotic surgery – a pilot scheme in the United Kingdom

**DOI:** 10.1186/s12909-025-07749-9

**Published:** 2025-08-08

**Authors:** Mohammed Al-Ani, Samuel Massias, Panduka Jayawardena, Esha Khanderia, Joshua Franklyn, Khawar Hashmi, Vanash Patel

**Affiliations:** 1 Department of Surgery, West Hertfordshire Teaching Hospitals NHS Trust, Watford General Hospital, Vicarage Road, Watford, WD18 0HB UK; 2 Department of Surgery and Cancer, Imperial College London, St Mary’s Hospital, 10th Floor QEQM Building, London, W2 1NY UK

**Keywords:** Robotic assisted surgery, Specialist training, Medical education

## Abstract

**Background:**

Training in robotic colorectal surgery is predominantly confined to senior trainees and consultants. The lack of robotic training of the general surgical specialist trainee (ST) in the formative stages of training has led to widespread discontent among them. This paper describes a pilot scheme to establish a robotic training programme for general surgical STs in a district general hospital.

**Methods:**

Six STs were enrolled between May 2023 and October 2023. A training programme was established using the CMR Surgical Versius robotic system, which consisted of online modules, a first assist course, performance-tracked virtual simulation, basic surgical skills assessment, and live operating. The programme was evaluated using the Royal College of Surgeons Participant Feedback Questionnaire (Likert scale 1–5).

**Results:**

Trainees ranging from ST4 to ST8 took a median of 4.5 days (IQR 2) to complete 16 performance-tracked virtual simulation tasks. There was a significant variance in the number of attempts to pass each task (*p* < 0.0001), with STs finding endoscope control, suturing and knot-tying the hardest tasks to pass. There was a significant variance between the STs in the number of attempts to pass all the tasks (*p* = 0.02), with the ST4 trainee performing significantly better than the ST7 and ST8 trainees (*p* < 0.05). The STs participated in 43 operations (anterior resection = 13, abdominoperineal resection = 4, right hemicolectomy = 10, loop colostomy = 1 and cholecystectomy = 15). There were no complications related to training. Feedback on the programme was positive with a median score of 5 (IQR 0.75) for all sections of the questionnaire.

**Conclusions:**

A structured robotic training programme to democratise robotic surgical training, especially among junior trainees who may not have prior laparoscopic surgical experience is safe and feasible. Replicating similar schemes in other hospitals may improve robotic access and training for STs.

## Background

Robotic-assisted surgery (RAS) is perceived to have distinct advantages over conventional laparoscopic surgery [1, 2]. This has resulted in its expansion over recent years, with the American College of Surgeons predicting a transformational shift towards RAS [3]. If present trends continue, robotic surgery is likely to surpass conventional laparoscopic and open surgery as the preferred surgical approach by the year 2025; however, the training pathways to achieve competence in RAS have been rudimentary and have not kept pace with the times [4–6]. At present, there are predominantly two approaches to training surgeons in RAS. Consultants are trained by other established consultants via the proctorship pathway, and senior fellows (who have completed their formal training) take on fellowships to obtain competence [7, 8]. There is a hiatus in the training of trainees during their formative years, resulting in widespread discontent and frustration among this cohort [9]. The lack of a streamlined training pathway has also been highlighted by the Royal College of Surgeons and the European Society of Coloproctology, who have issued guidance to help establish robotic training pathways for all trainees, not just for senior trainees and consultants [7, 8]. It is currently recognised that > 70% of surgical trainees in the UK and Ireland across all specialities have no access to robot-assisted surgical training [10]. Additionally, a review identified specific needs for a robotic faculty development curriculum which included maximizing dual-console (or open console) teaching, theatre team training, non-technical skills training, patient safety, user-machine interface training and telementoring [11].

In this context, a pilot scheme was introduced at West Hertfordshire Teaching Hospitals NHS Trust (WHTH) to establish a standardised curriculum for robotic training for trainees in general surgery, which could be integrated into the current surgical training curriculum. In this paper, we outline how the pilot scheme was set up and report on the feedback from trainees regarding their experiences of the training pathway.

## Methods

Robotic colorectal, upper gastrointestinal, and general surgery using the CMR Surgical Versius robotic system was introduced at WHTH in July 2022. All consultant trainers underwent robotic training via the ‘Train the Trainer’ pathway provided by CMR Surgical [12]. This programme, together with the wider CMR Surgical educational platform, has been accredited by the Royal College of Surgeons of England [13]. Once consultants had completed their learning curve with the Versius system, they were approved to train surgical trainees.

A structured pilot programme was subsequently developed to train specialist trainees (ST4–ST8) in robotic console surgery using Versius. Trainees who previously would have been trained laparoscopically were now enrolled into this pilot scheme. Trainees self-nominated for participation based on a stated interest in robotic surgery and were selected from the existing general surgery rotation at WHTH. All had no prior robotic console experience and were scheduled to rotate through WHTH, ensuring compatibility with their standard clinical duties.

**Fig. 1:**
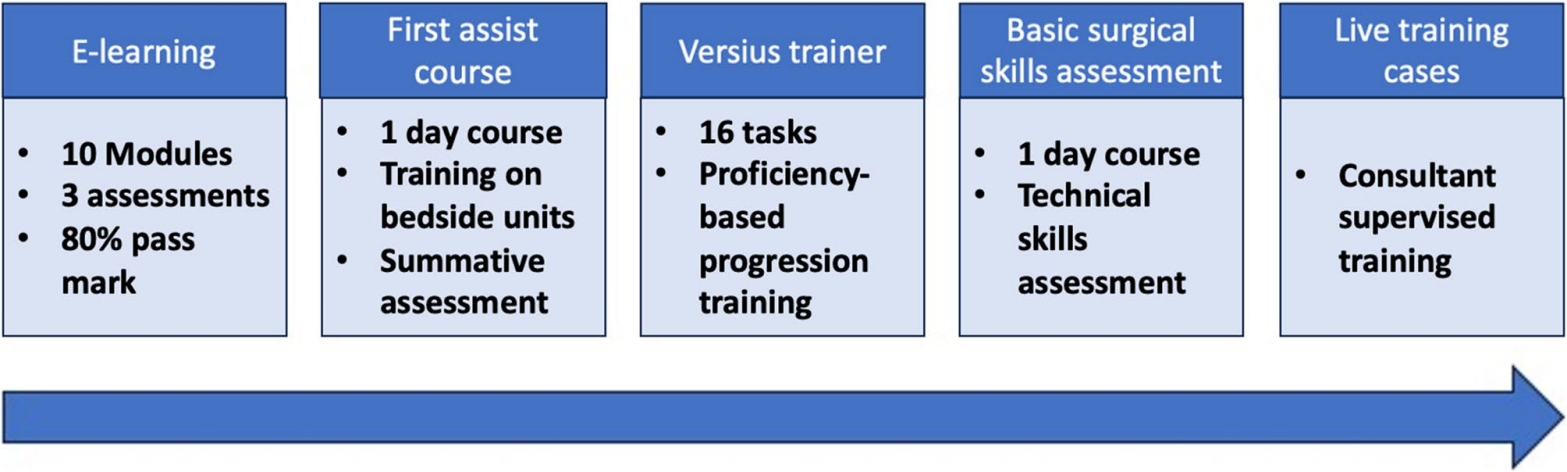
Components of the robotic training programme for trainees

**Fig. 2:**
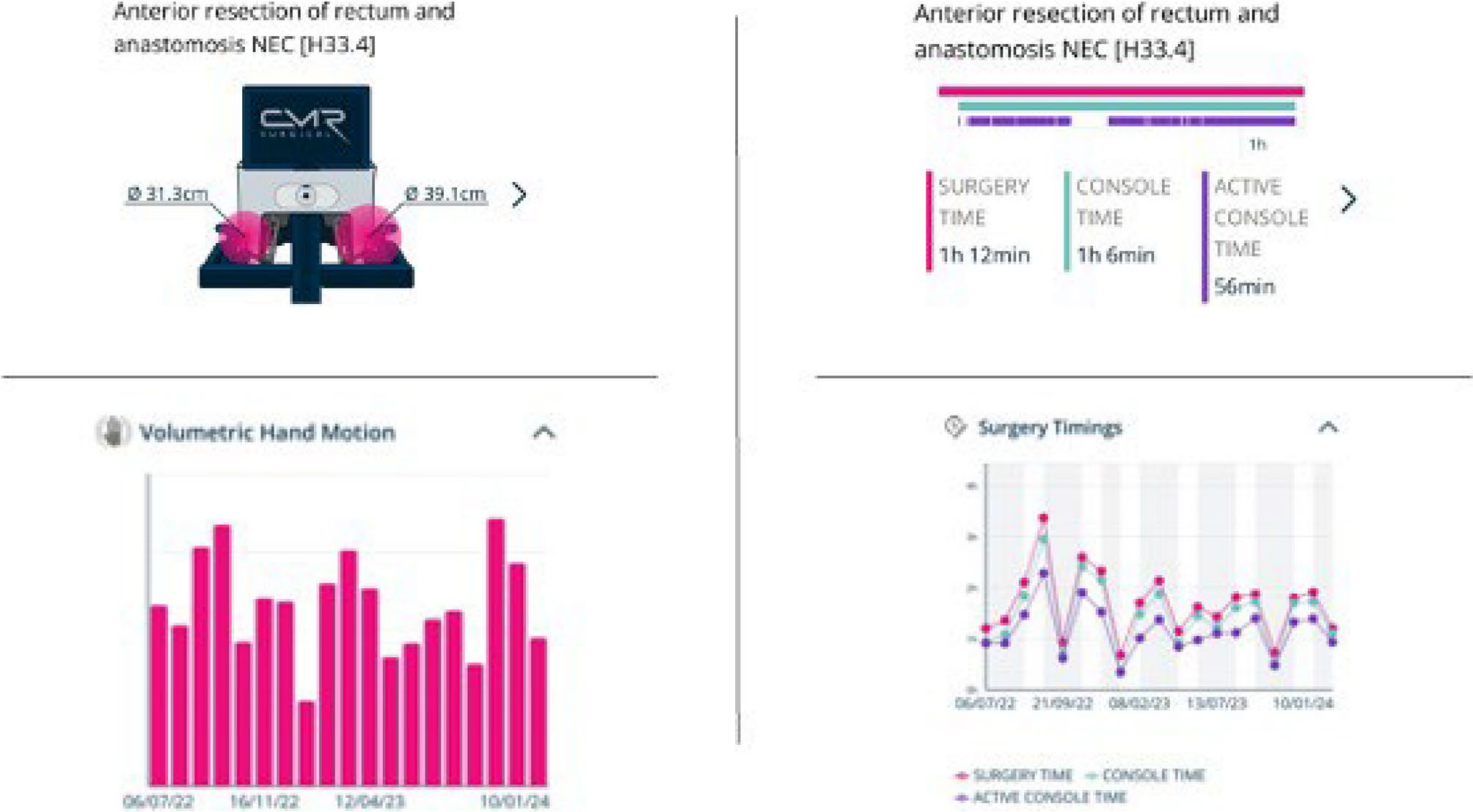
Examples of feedback data from robotic surgery using Versius

The training programme incorporated the validated consultant proctorship pathway and included sequential steps: online didactic modules, a first-assist course, performance-tracked virtual simulation exercises using the Versius Trainer, basic surgical skills assessment, and supervised training in live operating lists (Fig. 1). Progression through the pathway was contingent on meeting defined benchmarks at each stage, using objective performance metrics. Consultant trainers employed training principles similar to laparoscopic instruction, supplemented by metrics such as overall surgical time, active console time, and volumetric hand motion data to guide mentoring (Fig. 2).

Trainees did not receive dedicated time off from service for training. Instead, the programme was embedded into their clinical schedule, with specific course days approved by the rota coordinator and educational supervisor as study leave. E-learning and simulation components were designed to be self-directed and completed during protected time or when clinical duties allowed, ensuring service provision remained unaffected while supporting skill acquisition.

### Training pathway

#### Step 1: E-learning modules

Trainees had to complete 10 e-learning modules with 3 additional assessment modules and a score of 80% was essential to pass the modules. A list of the modules and assessments is outlined in Table 1.

**Table 1:** E-Learning modules and assessments for robotic training

Pre-operative Phase	
Module 1	Introduction to the Versius Surgical System
Module 2	Pre-Operative - The Surgeon Console
Module 3	Pre-Operative - Versius Bedside Units
Module 4	Pre-Operative - Draping the Bedside Units, Camera Head and Reviewing the Checklist
Module 5	Pre-Operative - Handing Control to the Surgeon Console
Assessment 1	Pre-Operative Phase
Intraoperative Phase	
Module 6	Using the Instruments and Endoscope During Surgery
Module 7	Using Surgical Energy with the Versius Surgical System
Module 8	Managing the Bedside and Hand Controller Use
Assessment 2	Intra-Operative Phase
Post-operative Phase	
Module 9	Post-Operative Workflow
Module 10	Managing Alarms and Troubleshooting
Assessment 3	Post-Operative Phase

#### Step 2: first assist course

Trainers from the CMR Surgical professional educational team held a one-day in-person training course for participating trainees. This taught them how to set up Versius as a team, drape the bedside units aseptically, respond appropriately to various alarms and safety features, provide bedside assistance, and clean the equipment. Following this, there was a summative assessment that candidates completed to ensure learning objectives had been achieved.

#### Step 3: versius trainer

The training on the Versius trainer consists of 16 tasks and is proficiency-based (Table 2). Each task has measurable metrics such as the combined instrument tip path length to reaching the object, combined instrument angular path and combined instruments out of view. The performance in each of these metrics is given a score, these performance scores are compared with benchmarks set to the average performance of experts completing the training tasks [14].

**Table 2:** Training tasks

Tasks marked with * must be passed 2 times consecutively and 3 times in total. All other tasks must be passed once	
1	Instrument navigation
2	Jaw control
3	Endoscope control *
4	Multitarget *
5	Four arms
6	Electrosurgery *
7	Peg transfer *
8	Letter manipulation *
9	Pattern cutting circle
10	Pattern cutting star *
11	Wire loop 1
12	Wire loop 2
13	Blunt and sharp dissection
14	Needle driving *
15	Stitch and square knot *
16	Running suturing

#### Step 4: basic surgical skills assessment

**Fig. 3:**
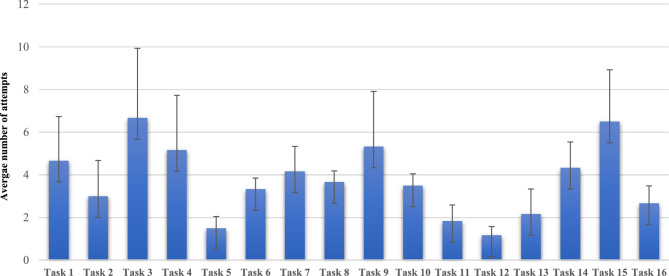
Graph to show the average number of attempts to complete the Versius Trainer tasks

The basic surgical skills assessment was an in-person dry box session in the laboratory using synthetic models shown in Fig. 3. The aim of this was to develop trainees’ orientation and performance-based metrics. Trainees undertook technical skills-based assignments following a video demonstration and received supervisor feedback during and after the task. The tasks were designed to test the robotic system’s full range of motion and capabilities. These covered 9 types of dissection, suturing, knot-tying and a 360-degree anastomosis. Candidates were scored from analysis of technique and event errors and had unlimited attempts to pass the assessment.

#### Step 5: live training cases

Trainees subsequently participated in supervised training sessions under the direct guidance of consultant trainers, who provided real-time oversight during robotic procedures. Each operation was deconstructed into key procedural steps, allowing trainees to focus on mastering individual components before progressing to full procedural integration. For instance, anterior resections were taught using a modular approach based on established laparoscopic training frameworks. This included medial-to-lateral dissection, vascular control (ligation of the inferior mesenteric artery and vein), lateral mobilisation of the sigmoid colon, and total mesorectal excision. These structured steps were adapted from conventional laparoscopic paradigms and incorporated into the robotic training curriculum to facilitate progressive and systematic skill development. The modular format also allowed case segmentation based on the trainee’s competence level, ensuring patient safety during early robotic experience. This immersive, structured training approach enabled trainees to safely and effectively develop proficiency as robotic console surgeons [15].

At the end of the course, the trainees completed participation feedback forms, one for the first assist course and another for the basic surgical skills assessment. The questionnaire used was obtained online via the Royal College of Surgeons (RCS) website [16]. This was an RCS-developed questionnaire with the items in the questionnaire reflecting the College’s Education Standards.

### Statistical analysis

One-way analysis of variance (ANOVA) was used to assess differences between tasks and trainees on the Versius Trainer. Data were analysed using IBM SPSS Statistics (Version 29).

## Results

Between May 2023 and October 2023, six trainees were enrolled on the pilot training scheme, and five consultant surgeons trained them. Trainees ranged from specialist trainee level 4 to level 8 and all trainees completed the training pathway and were able to train as console surgeons on live cases (Table 3). With regards to the performance-tracked virtual simulation exercises using Versius Trainer (Step 3), trainees required a median of 4.5 days (IQR 2) to complete all components of the training programme. These were not taken as consecutive days but were flexibly scheduled around clinical responsibilities, based on individual availability and service demands. The number of attempts for each trainee to complete each task is shown in Fig. 3.

**Table 3:** Participant workflow and resource allocation

Phase	Time per Trainee	Key Resources
E-learning	2 weeks	Online platform
First Assist Course	1 day	Trainer, robotic system
Virtual Simulation	4.5 days (median)	Versius Trainer, supervision
Basic Skills Assessment	1 day	Dry lab models, faculty feedback
Live Cases	3–6 months	Theatre time, consultant supervision

There was a significant variance in the number of attempts to pass each task (*p* < 0.0001), with STs finding endoscope control, suturing and knot-tying the hardest tasks to pass. There was a significant variance between the STs in the number of attempts to pass all the tasks (*p* = 0.02), with the ST4 trainee performing significantly better than the ST7 and ST8 trainees (*p* < 0.05).

**Fig. 4:**
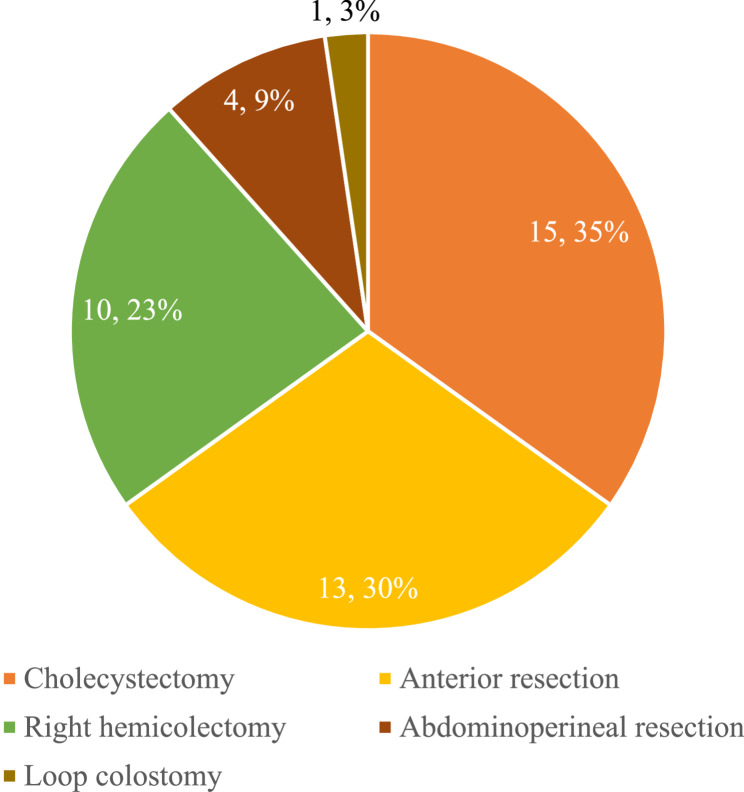
Type of operations in which trainees participated as console surgeons

Trainees participated in 43 operations as console surgeons which included right hemicolectomy, anterior resection, abdominoperineal excision, loop colostomy and cholecystectomy. The numbers of cases and breakdown are shown in Fig. 4. No adverse events related to the trainee were reported in the training cases. Feedback from consultant trainers was conveyed verbally and documented through work-based assessments on the Intercollegiate Surgical Curriculum Programme logbook.

### Feedback from the trainees

All six candidates who completed the training filled out the RCS-developed questionnaires for both the first assist course and the basic surgical skills assessment. For general administration, both the first assist course and basic surgical skills assessment averaged a score of 4.7 out of 5.0 (94%). The content and delivery scored an average of 5.0 out of 5.0 (100%), and the learning outcomes averaged 4.8 out of 5.0 (96%) for both educational modalities. Finally, the course recommendations scored 4.9 out of 5.0 (98%) for the first assist course and 5.0 out of 5.0 (100%) for the basic surgical skills assessment. The feedback comments given by the trainees for both educational modalities were entirely positive. For the first assist course they included: “one to one training on the day”, “calm environment, which encouraged learning” and “very good for transferring skills to actual operating on patients using console”. For the basic surgical skills assessment, these included: “small training groups, with good trainer ratio”, “very well explained and elaborated” and “hands-on experience and lots of practice with setting up robotic arms”.

## Discussion

This pilot scheme demonstrates the feasibility of providing structured robotic training to surgical trainees and outlines a standardised framework that can be used across hospitals that use the Versius robotic system in the United Kingdom (UK). The training initiative at WHTH demonstrates that training the trainees in RAS can be implemented within a year of consultants starting their own RAS training. This timeframe is pragmatic, considering the substantial volume and exposure needed to attain the required level of expertise using robotic systems. The exact learning curve for robotics training is not well-defined and learning curve estimates are subject to considerable uncertainty [17]. As a direct consequence of the pilot scheme, centralised trainee feedback to the deanery regarding trainee experience at WHTH improved and the hospital was overwhelmed with trainees who wanted to rotate through WHTH to gain exposure to robotic training. This level of enthusiasm may reflect the current lack of access to robotic training opportunities across the UK and Ireland, where over 70% of surgical trainees report no exposure to such training. This highlights both the relevance and potential impact of initiatives like our pilot programme in addressing this significant gap [10].

The influx of newer technology by different surgical companies brings unique challenges to the surgical education landscape. In the UK, at present most robotic curricula are designed by the industry to upskill fully trained surgeons in using newer technology. This consultant-centric training model has changed the intraoperative experience of trainees, resulting in an indifferent or discouraging learning environment for them [18, 19].

The primary barrier to accessing robotics as a trainee is the lack of a formal training pathway [8]. This principle is supported by the FOS: TEST report (2022), which identifies the lack of exposure to robotic surgery as an ‘unmet need’ for trainees and recommends the implementation of a core robotics curriculum as a potential intervention [20]. Streamlined training pathways are prerequisites to having sustainable training, otherwise it is easy for the consultants to be more mindful of their own training needs at the expense of surgical training [21, 22]. This pilot scheme shows that within a year of starting robotic surgery, the consultants were able to safely train the trainees as robotic console surgeons. Establishing a robotic training programme is essential as this enables trainees who are in rotation to neatly slot into the pathway and thereby expect to have hands-on console training in due course.

Whilst having a robotic training pathway is essential, it is equally important for the relevant training stakeholders (consultant surgeons, deanery representatives and industry) to be invested in the training of trainees [23]. The cost of participation per trainee was approximately £1,500, covering access to the Versius Trainer, course materials, and simulation resources. This was fully reimbursed by the local postgraduate deanery, eliminating financial burden on trainees. Additional training components, including online modules and the First Assist Course, were subsidised by CMR Surgical through an educational partnership. Consultant trainers contributed under their existing NHS contracts without additional remuneration, with training duties integrated into their clinical and educational roles. The programme was therefore resource-efficient, leveraging institutional support, industry collaboration, and deanery funding to minimise financial and logistical barriers. It is tempting to focus resources on senior trainees or consultants, the true scope and implementation of robotics can only be fulfilled if a ‘ground-up’ training model is pursued. Financial and educational support through the Deanery, individual hospitals and industry is integral to the success of robotic training. In the case of this pilot scheme funding was obtained via the local deanery, the parent hospital and industry to ensure that all trainees had access to robotic training. In this pilot, funding from the local deanery, parent hospital, and industry enabled trainee participation, though access was limited to six STs due to resource constraints. The model offers a scalable framework for broader implementation with institutional support.

Whilst this paper discusses a robotic training pathway for trainees in a teaching hospital, in the long run, the surgical curriculum would need a pragmatic overhaul to incorporate robotic training into the certification process [9, 24]. Pathways that ensure trainees are safely introduced to new technology are vital; these pathways need to culminate in appropriate credentialing of the trainees (certification of completion of training) [5, 25].

The feedback received from the participants in the pilot project has been one of overwhelming enthusiasm and all trainees would recommend this pilot scheme to their peers. Future modifications to further improve access and affordability include the use of VR headsets where trainees can access device training and basic robotic skills training in their own time at home [26]. Despite the overall positive feedback from the current curriculum, there are certain limitations that we must recognise. This scheme has been rolled out in a single centre using a single robotic platform, this could potentially limit its generalisability. Additionally, the small sample size is a limitation that we acknowledge. However, to our knowledge, this is the first paper to address the issue of robotic training in formative years of surgical training in the UK and the results of this pilot project are encouraging.

## Conclusions

A structured robotic training program to democratise robotic surgical training for trainees, especially among junior trainees who do not have prior robotic surgical experience is safe and feasible. We hope this pilot scheme will encourage other hospitals to set up their robotic training pathways to increase access to robotic surgical training for trainees in the UK.

## Data Availability

No datasets were generated or analysed during the current study.
